# Inter‐annual variation in the abundance of specialist herbivores determines plant resistance in *Datura stramonium*

**DOI:** 10.1002/ece3.10794

**Published:** 2023-12-06

**Authors:** Ivan M. De‐la‐Cruz, Juan Núñez‐Farfán

**Affiliations:** ^1^ Laboratory of Ecological Genetics and Evolution, Department of Evolutionary Ecology, Institute of Ecology Universidad Nacional Autónoma de México Mexico City Mexico; ^2^ Department of Plant Protection Biology Swedish University of Agricultural Sciences Alnarp Sweden

**Keywords:** herbivory, phenotypic selection, plant resistance, relative growth rate, resource allocation

## Abstract

The expression of plant resistance traits against arthropod herbivores often comes with costs to other essential plant functions such as growth and fitness. These trade‐offs are shaped by the allocation of limited resources. However, plants might also possess the capability to allocate resources to both resistance and growth, thereby ensuring their survival when under herbivore attacks. Additionally, the extent of damage caused by herbivores could vary across different years or seasons, subsequently impacting plant performance. In this study, we aimed to investigate how the annual variations in herbivore abundance and damage levels affect plant performance. We generated F_2_ progeny through a cross between two populations of the annual herb *Datura stramonium* (Solanaceae). These populations are known to have differing levels of chemical defense and herbivory. These F_2_ plants were cultivated in a common natural environment for two consecutive years (2017 and 2018). Our findings reveal that plants with higher resistance, attained higher seed production but this trend was evident only during 2018. This relationship coincided with a five‐fold increase in the abundance of *Lema daturaphila* (Chrysomelidae) larvae in 2018. Indeed, the plants experienced a 13‐fold increase in damage during this second year of study. Furthermore, our results indicated that there was no trade‐off between resistance, growth, and fitness in either of the 2 years. In contrast, during 2018, when plants faced stronger herbivore pressure, they allocated all available nutritional resources to enhance both resistance and growth. Our study highlights how the selection for plant resistance is dependent upon the inter‐annual variation in herbivore abundance.

## Graphical abstract

The study underscores the influential role of specific herbivores in driving plant resistance and offers fresh insights into the inter‐annual evolutionary dynamics of plant resistance.

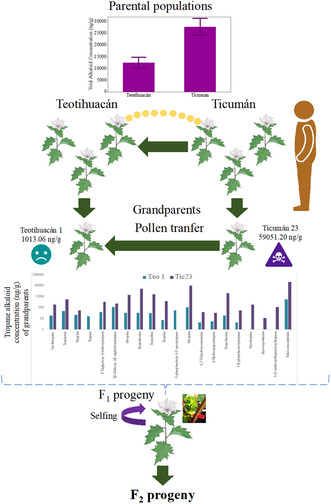

## 1 INTRODUCTION

Plants face the challenge of being attacked by multiple species of herbivores either simultaneously or sequentially. This, in turn, drives natural selection on their defensive traits, encompassing both indirect and direct defenses, with the aim of minimizing damage (Agrawal et al., 2012; Thompson, 2005; Wise & Rausher, 2013). The presence of multiple herbivores attacking a plant can significantly alter the evolutionary dynamics of plant defense against herbivory (Edwards et al., 2023; Wise & Rausher, 2013, 2016). However, due to variations in the extent of damage caused by different herbivores, plants must strategically determine how to allocate their limited nutrient resources to costly defensive traits, in order to effectively manage diverse herbivore pressures (Schuman & Baldwin, 2016; Wise & Rausher, 2016; Züst & Agrawal, 2017). As a result, plants may allocate nutrient resources to preferentially defend themselves against one herbivore species, which consequently reduces defense against another herbivore species (known as diffuse coevolution; Iwao & Rausher, 1997; Juenger & Bergelson, 1998; Wise & Rausher, 2013). Additionally, variations in herbivore abundance, such as insect outbreaks, occurring between seasons or years, can also impact the selective pressure on plant defense (as reviewed by Agrawal & Maron, 2022). For instance, research indicates that certain genotypes possessing specific chemical defenses exhibit higher survival rates during intense insect herbivore attack compared to those genotypes that lack such chemical defenses (Züst et al., 2012). Consequently, outbreaks of herbivores have the potential to induce alterations in population genetic composition and demography (Agrawal & Maron, 2022). Therefore, it is essential to determine whether natural selection acting on plant defense varies over time (e.g., between years), in order to gain a comprehensive understanding of the evolutionary dynamics between plants and herbivores, particularly within the context of changing environments.

The production of defense traits in response to different herbivores can result in increased costs of resistance (often quantified as the inverse of herbivore consumption; Simms & Rausher, 1987) and potential trade‐offs with other plant functions, such as growth and reproduction (e.g., allocation cost; He et al., 2022; Herms & Mattson, 1992; Monson et al., 2022; Watts et al., 2023; Züst & Agrawal, 2017). Any redirection of limited nutrient resources from primary metabolism to defense mechanisms may lead to a reduction in growth and reproduction (Züst & Agrawal, 2017). In contrast, when nutrient resources are not a constraint (e.g., rich‐nutrient soils), it is likely that trade‐offs between growth and defense may disappear or at least be reduced. This could lead to plants simultaneously growing and defending (He et al., 2022; Monson et al., 2022). Empirical evidence also suggests that the relationship between growth and defense can vary depending on factors like the timing and level of damage received by plants and the abundance of herbivores present (Mauricio et al., 1997; Strauss & Agrawal, 1999; Züst & Agrawal, 2017). For example, the annual variability in insect herbivore abundance, such as insect outbreaks, is expected to increase plant damage and ultimately affect natural selection on plant resistance and growth (Agrawal & Maron, 2022). Furthermore, evidence indicates that some herbivores tend to prefer larger plants due to their higher biomass for feeding (Cornelissen et al., 2008; Price, 1991; Schlinkert et al., 2015; White, 1969). As a result, plants must strategically allocate their available resources to either defensive traits and/or growth to survive herbivore attacks. However, there is still a scarcity of empirical studies investigating the inter‐annual effects of herbivory on plant growth and fitness, as well as the potential trade‐offs across years (Agrawal & Maron, 2022; De Jong & Van Der Meijden, 2000; Valverde et al., 2003; Züst & Agrawal, 2017).

The main aim of this study was to assess the effects of herbivory (including both the damage inflicted and the abundance of multiple herbivores) and relative growth rate (RGR) on plant fitness across multiple years (2017 and 2018). Additionally, we aimed to identify potential trade‐offs among plant resistance, RGR, and reproduction. We hypothesize that plants with higher fitness will have an increased resistance and a lower number of herbivores over the 2 years. This hypothesis suggests that plant resistance traits (e.g., secondary compounds and trichomes) reduce herbivory, thereby conferring a fitness benefit to the plants. Additionally, we propose that larger plants with faster growth rates will show higher fitness but lower resistance to herbivores. This second hypothesis posits that plants preferentially allocate nutrients towards rapid growth to accelerate their life cycle and secure reproductive success, rather than allocating resources to the production of costly resistance traits. To achieve this, we generated F_2_ progeny by crossing two distinct parental plants from different populations of the annual herb *Datura stramonium* (Solanaceae). These populations exhibit variations in chemical defense levels and herbivore communities (De‐la‐Cruz, Cruz, et al., 2020; De‐la‐Cruz, Merilä, et al., 2020). The F_2_ plants were planted in a common natural environment in Mexico for both consecutive years. Our investigation focused on two main questions: (1) Does the relationship between sexual fitness and plant resistance, herbivore abundance and RGR vary between years? (2) Are there trade‐offs between resistance, RGR, and fitness across different years?

## 2 MATERIALS AND METHODS

### 2.1 The study system

*Datura stramonium* is an annual herb that only grows during the summer season in Mexico (June–September; Núñez‐Farfán & Dirzo, 1994). This species produces erect fruits and is distributed throughout North and South Mexico (Bye & Sosa, 2013; Mace et al., 1999). This species is well‐known for its highly toxic tropane alkaloids and terpenoids against insect herbivores (Castillo et al., 2015; De‐la‐Cruz, Merilä, et al., 2020; Miranda‐Pérez et al., 2016). It has been discovered that foliar trichomes also play a role in the defensive mechanisms of *D. stramonium* (Valverde et al., 2001). Previous studies have documented the evolution of certain tropane alkaloids and terpenoids through positive selection by herbivores (Castillo et al., 2015; De‐la‐Cruz, Merilä, et al., 2020; Miranda‐Pérez et al., 2016; Shonle & Bergelson, 2000).

The experimental site, situated in Teotihuacán (State of Mexico, coordinates 19°41′6.96″ N, 98°52′19.63″ W), was selected due to the presence of three specialist herbivore species that infest *D. stramonium*. These herbivores consist of the two chewing beetles *Lema daturaphila* (Chrysomelidae), *Epitrix parvula* (Chrysomelidae), and the seed predator *Trichobaris soror* (Curculionidae) (De‐la‐Cruz, Merilä, et al., 2020). Evidence indicates that these three main herbivores are present across nearly the entire geographical range of *D. stramonium* in temperate climates, with their development being closely linked to the growing season of *D. stramonium* (Castillo et al., 2013, 2015; Miranda‐Pérez et al., 2016; Núñez‐Farfán & Dirzo, 1994). The abundance of these herbivores fluctuates over time in the Teotihuacán site. Furthermore, prior research has demonstrated that these herbivore species exert selection pressures for increased resistance in *D. stramonium* at this site (Carmona & Fornoni, 2013; De‐la‐Cruz, Merilä, et al., 2020).

### 2.2 Experimental design

**FIGURE 1:**
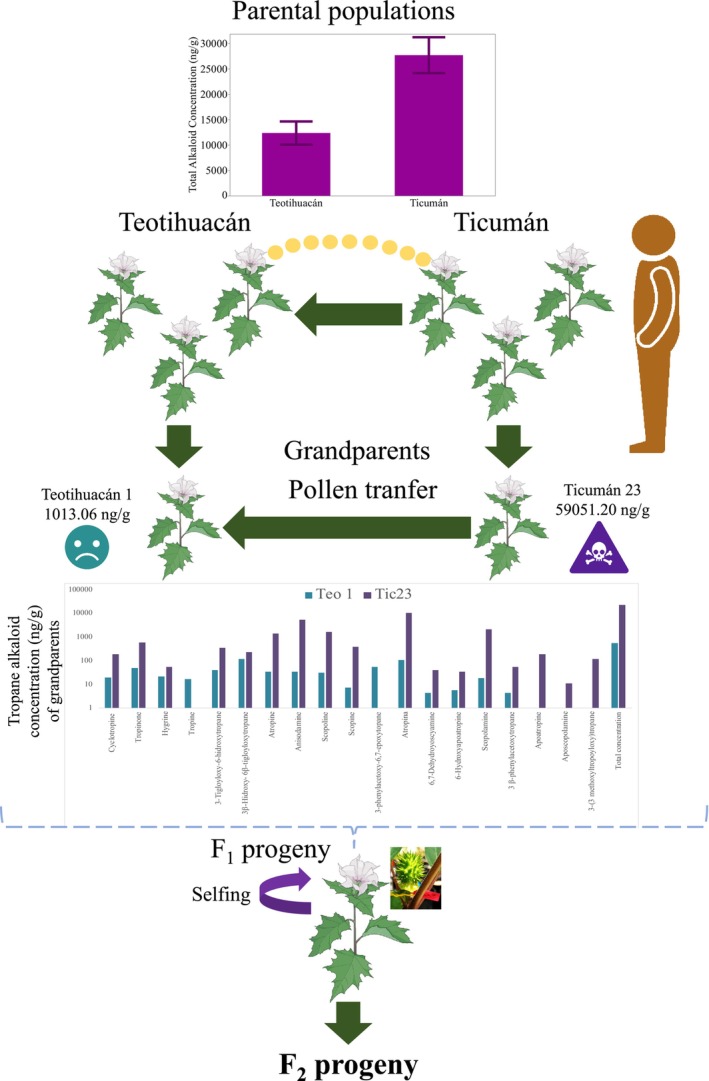
Depiction of the experimental design used to produce the F_2_ generation progeny used in the study. Random crosses (ca. 200) were carried out with plants from Ticumán and Teotihuacán (Parental populations). Plants from these two populations are highly differentiated in their concentration of tropane alkaloids. We screened the concentration of 21 tropane alkaloids for each plant. From all the crosses, we selected the couple with the most differentiated individuals in their concentration of tropane alkaloids; Ticumán 23 and Teotihuacán 1. This cross was self‐pollinated to generate a F_1_ progeny. Seeds from the F_1_ progeny were germinated, and we only selected one individual to produce seeds (F_2_ progeny). F_2_ seeds were germinated, and seedlings were transplanted to a common environment in Teotihuacán where the main specialist herbivores of *Datura stramonium* occur (see Section 2 for details).

For the production of the F_1_ and F_2_ progeny used in this study, a total of 21 tropane alkaloids were initially identified and analyzed for the parental plants (comprising 45 and 47 distinct plants from Teotihuacán and Ticumán, respectively), employing the methods outlined in De‐la‐Cruz, Merilä, et al. (2020) (see also Figure 1). Briefly, to extract the tropane alkaloids from each plant, frozen leaf tissue was transferred to 2‐mL Eppendorf tubes, grinding it with a plastic pestle while keeping it frozen by adding liquid nitrogen (De‐la‐Cruz, Merilä, et al., 2020). We then weighed the pulverized frozen leaf tissue in Eppendorf tubes. We added two steel balls to each Eppendorf tube along with 1.5 mL of extraction buffer (80% methanol; MeOH; and 1% formic acid); the tubes were then shaken for 60 s at 30 Hz in a TissueLyser II (QIAGEN Inc.) (De‐la‐Cruz, Merilä, et al., 2020). Finally, the samples were centrifuged for 20 min at 14,000 revolutions per minute; 700 μL of supernatant was collected and stored in glass vials (1.5 mL) and maintained at −4°C (De‐la‐Cruz, Merilä, et al., 2020). The samples were injected into an Agilent 1260 Infinity, coupled to an Accurate‐Mass Time‐of‐Flight (TOF) LC/MS‐6230, with an auto‐sampler Agilent Technology 1200 Infinity (De‐la‐Cruz, Merilä, et al., 2020).

The cumulative quantity of all alkaloids represented the overall tropane alkaloid concentration of each parental plant (Figure 1). Subsequently, we selected the pair displaying the greatest disparity in total tropane alkaloid concentration: the parents Teotihuacán 1 and Ticumán 23 (Figure 1). These plants exhibited a 58‐fold difference in their total alkaloid concentration (1013 vs. 59,000 ng/g of leaf, respectively) (Figure 1). As the plants reached the flowering stage, we carried out manual pollination of the flowers. The parent plant from Teotihuacán served as the pollen receptor, while the parent from Ticumán acted as the pollen donor (De‐la‐Cruz, Cruz, et al., 2020; De‐la‐Cruz, Merilä, et al., 2020; Figure 1). Germinated F_1_ seeds resulting from the cross between these two parental plants were cultivated and grown, following the procedure detailed in De‐la‐Cruz, Cruz, et al. (2020) and De‐la‐Cruz, Merilä, et al. (2020) (but see Figure 1). To germinate the F_1_ progeny, we utilized seeds from three fruits of the same selected cross (Teotihuacán 1 and Ticumán 23) (Figure 1). Among the germinated F_1_ plants (*n* = 8), we randomly selected a single plant, whose flowers were enclosed in bags to prevent pollen contamination from other plants (although the plants were cultivated in a glasshouse; Figure 1). This particular F_1_ individual was allowed to self‐pollinate to generate the F_2_ generation progeny (single‐family: Figure 1).

The seeds from the parental plants, F_1_, and F_2_ progenies were germinated by immersing them in water containers and maintaining them within an environmental chamber under a photoperiod of 12:12 L:D, at a temperature of 30°C during the day and 25°C at night, with a constant humidity of 85%. To promote germination, the seeds were sacrificed (Fornoni & Núñez‐Farfan, 2000). Subsequently, the germinated F_2_ seeds were transplanted into plastic pots (237 mL) filled with a 1:1 mixture of sand and vermiculite, and then randomly distributed across benches within the greenhouse. Each F_2_ plant received a uniform daily water supply (500 mL) until they were relocated to natural conditions. F_2_ seeds were germinated separately for each year of study.

### 2.3 Field experiment

#### 2.3.1 Experiment design

Once the two true leaves had emerged, F_2_ seedlings (*n* = 230) were transplanted at the start of June in both 2017 and 2018, enabling the F_2_ plants to experience the natural conditions of the experimental site in Teotihuacán. The planting of F_2_ seedlings followed a complete randomized design, with plants being spaced 1 m apart in a uniform grid pattern. The experimental plot was regularly weeded to prevent interference and competition by other species.

#### 2.3.2 Damage by herbivores

The percentage of consumed leaf area by the three main chewing herbivores was estimated with the mobile application BioLeaf (Machado et al., 2016) during three sampling periods (15, 30, and 45 days after planting) in each year. We took photographs of eight randomly chosen fully expanded leaves per plant using a mobile phone (Samsung Galaxy S6 edge) in each sampling date. The app automatically calculates the injured leaf regions caused by insect herbivory and then estimates the defoliation (in percentage) relative to the total leaf area (Machado et al., 2016). Thus, we estimated the average damage during the three sampling periods per plant. However, it is important to point out that in 2018 most leaf tissue was completely eaten by herbivores in many plants. In these cases, we assigned 100% of the damage to these plants.

#### 2.3.3 Herbivore abundance

Three species of herbivores were recorded during three sampling periods (15, 30, and 45 days after planting). In each plant, we counted the adults of (1) *Epitrix parvula*, (2) *Lema daturaphila*, and (3) *Trichobaris soror*. We also counted the abundance of larvae of (1) *Lema daturaphila*, and (2) *Trichobaris soror* (visible inside the fruits). Since larval development and pupation of *E. parvula* occur in the soil, we were unable to record these stages. Therefore, only the number of adults of this herbivore species per plant was recorded. To minimize bias in insect counting, only one person counted the herbivores on each plant in all the sampling periods. At the end of the experiments, we summed the three sampling periods as a measurement of the total abundance that each plant experienced by each herbivore in both years.

#### 2.3.4 Plant performance

At the end of the experiment (2 months after sowing), we collected all fruits produced by each plant. Fruits were bagged individually and labeled. In the lab, seed set per fruit was counted and the total number of seeds per plant was used as a proxy of maternal plant fitness (see Section 2.4; Mauricio & Rausher, 1997; Motten & Antonovics, 1992; Nunez‐Farfan et al., 1996). The plant size (plant height; cm) of the plants was also measured during all sampling sessions. Plant height was scored from the base of the stem (at the soil surface) to the tip of the terminal bud using a measuring tape.

### 2.4 Statistical analysis

#### 2.4.1 Statistical considerations

All statistical analyses were performed using the JMP PRO package (v17.0; SAS Institute). Plotting was made using ggplot2 (Wickham, 2016) in R v1.1.463 (R Core Team, 2022).

First, standardized individual sexual fitness was calculated using the formula *w*_*i*_ = *X*_*i*_/$\bar{x}$, where *X*_*i*_ represents the total number of seeds produced per plant, and $\bar{x}$ denotes the average number of seeds per plant in the experiment for each year. We used the inverse of plant damage as a measure of plant resistance (calculated using the operational definition 1−mean proportion of leaf area damaged; Simms & Rausher, 1987). We calculated the Relative growth rate (RGR) as an indirect predictor of plant growth/resource acquisition (Camargo et al., 2015; Gianoli & Salgado‐Luarte, 2017) as follows:

$$\mathrm{RGR}=\frac{S2-S1}{T2-T1}$$

where S1 and S2 are the plant height measured at time 1 (T1; when plants were transplanted) and time 2 (T2; 45 days after transplantation 2), respectively.

#### 2.4.2 Inter‐annual effect on plant damage, fitness, relative growth rate, and herbivore abundance

We employed generalized linear models (GLMs) to examine variations in levels of leaf damage, fitness, relative growth rate, and herbivore abundance between the 2 years of study. For each of the response variables—fitness and herbivore abundance—we conducted separate GLMs using a Poisson error distribution with a log link function. A Student's *t*‐test was employed to assess the mean differences in relative growth rate between the 2 years under study. The normality of the residuals from the Student's *t*‐test analysis was assessed using the Shapiro–Wilk test (*W* = 0.99, *p* = .0863). Additionally, a GLM with a binomial distribution with a logit link was conducted to analyze leaf damage (response variable) variation between years. All models were performed using raw data.

#### 2.4.3 Relationship between plant fitness, resistance, and relative growth rate between years

We performed a Spearman's correlation analysis to assess the relationships among the damage, RGR, and abundance of each herbivore species for each year. All variables were standardized ($\bar{x}$ = 0, SD = 1). *p*‐values of the correlations were adjusted using the Benjamini–Hochberg False Discovery Rate (Benjamini & Hochberg, 1995). These correlations enabled us to examine which herbivore was most strongly positively correlated with plant damage. Additionally, we investigated whether larger and faster‐growing plants or smaller and slower‐growing plants were associated with higher herbivore abundance and damage.

A phenotypic selection analysis was then performed to evaluate the combined effect of RGR and resistance on plant fitness for each year (Lande, 1979; Lande & Arnold, 1983). For these analyses, we utilized a Poisson distribution with a log link. The relative sexual fitness was used as a response variable, while RGR, resistance, and their interaction were considered as predictors. By introducing the interaction term between resistance and RGR, we were able to investigate whether the relationship between resistance and sexual fitness is influenced by the plants' growth rate. This also allowed us to assess potential trade‐offs between plant performance and resistance.

We performed two additional GLMs (one per year) using a Poisson distribution with a log link. These models were constructed with fitness as the response variable and the total abundance of each folivore species, RGR, and their interaction as predictors. An additional GLM was conducted (Poisson distribution, log link) to assess the direct impact of the seed predator (*T. soror* larvae) on sexual reproduction. In this model, fitness was used as the response variable, while the abundance of *T. soror* larvae, RGR, and their interaction as predictors. Incorporating the interactions between RGR and herbivore abundance into our models, allowed us to investigate if relative plant fitness was affected by the interaction between growth and herbivore abundance.

All numeric variables used as predictors in the GLMs were standardized to a mean of zero and a standard deviation of one ($\bar{x}$ = 0, SE = 1). The model coefficients (also named the selection gradients; *β*_*i*_, Lande & Arnold, 1983) obtained from the models represent the strength and direction of predictors acting directly on plant fitness in comparable units (standard deviations; Wise & Rausher, 2013).

## 3 RESULTS

### 3.1 Inter‐annual effect on plant damage, fitness, RGR, and herbivore abundance

**FIGURE 2:**
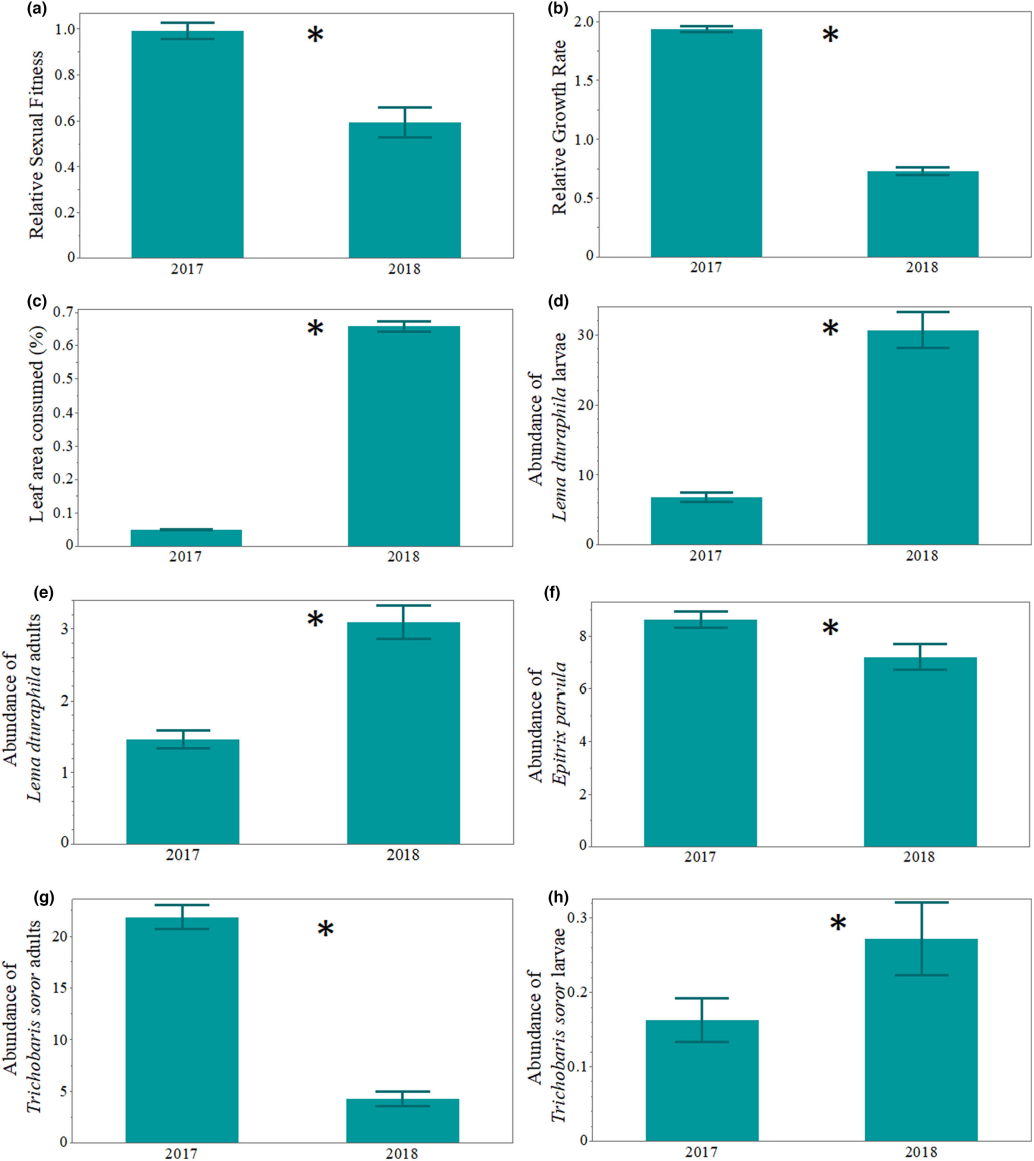
Barplots showing the mean differentiation and error bars of relative sexual fitness (a), relative growth rate (b), leaf area consumed % (c), abundance of *Lema daturaphila* larvae (d), abundance of *L. daturaphila* adults (e), abundance of *Epitrix parvula* (f), abundance of *Trichobaris soror* adults (g) and abundance of *T. soror* larvae (h) between the 2 years of study (2017 and 2018). Raw data were used for plotting. *Depicts a significant *p‐*value < .05. See also Table 1.

The percentage of leaf damage exhibited a significant 13‐fold increase in 2018 compared to 2017 (Table 1, Figure 2). The mean abundance of both adults and larvae of *L. daturaphila*, along with adults of *T. soror*, displayed significantly higher numbers in 2018 when contrasted with 2017 (Table 1; Figure 2). Conversely, the abundance of *E. parvula* adults and *T. soror* larvae was greater in 2017 (Table 1; Figure 2). A significant increase was observed in the relative growth rate and relative sexual fitness in 2017 compared to 2018 (Table 1, Figure 2).

**TABLE 1:** Mean differentiation of traits measured in this study between 2017 and 2018.

Year	*N*	Mean	SE	Estimate	SE	L‐R X^2^ or *F*	*p*
(a) Leaf area consumed							
2017	218	4.83	0.21	−1.79	0.17	184.05	**.0001**
2018	177	65.74	1.57				
(b) Relative growth rate							
2017	218	1.93	0.02	0.60	0.01	1031.48	**.0001**
2018	177	0.72	0.03				
(c) Relative sexual fitness							
2017	218	0.99	0.02	0.25	0.05	26.78	**.0001**
2018	177	0.59	0.02				
(d) Larvae of *Lema daturaphila*							
2017	218	6.79	0.71	−0.75	0.07	126.34	**.0001**
2018	177	30.63	2.55				
(e) *Lema daturaphila*							
2017	218	1.46	0.12	−0.37	0.05	44.88	**.0001**
2018	177	3.09	0.23				
(f) Larvae of *Trichobaris soror* (fruit)							
2017	218	21.79	1.17	0.81	0.11	87.39	**.0001**
2018	177	4.26	0.72				
(g) *Trichobaris soror* (leaf)							
2017	218	0.16	0.02	−0.25	0.12	4.21	**.0402**
2018	177	0.27	0.04				
(h) *Epitix parvula*							
2017	218	8.61	0.32	0.09	0.03	6.13	**.0132**
2018	177	7.18	0.48				

*Note*: (a) Leaf area consumed (plant damage). (b) Relative growth rate (RGR). (c) Relative sexual fitness. (d) Abundance of *Lema daturaphila* larvae. (e) Abundance of *Lema daturaphila*. (f) Abundance of *Trichobaris soror* larvae. (g) Abundance of *Trichobaris soror*. (h) Abundance of *Epitrix parvula*. Generalized linear models were performed using raw data.

Abbreviations: Estimate, coefficient of the model; L‐R X^2^, likelihood‐ratio test; *N*, number of individuals; SE, standard error. Significant *p*‐values are in bold.

### 3.2 Correlation between RGR and herbivory

In 2017, larger plants with a faster growing (higher RGR) had higher abundance of *T. soror* (both larvae and adults) and *E. parvula* (Appendix 1). In 2018, plants with higher damage (more susceptible) had higher abundance of *L. daturaphila* larvae (Appendix 1). Plants with lower damage (more resistant) had higher RGR values only in 2018 (Appendix 1).

### 3.3 Phenotypic selection analyses for plant resistance, RGR, and herbivore abundance

**FIGURE 3. 3:**
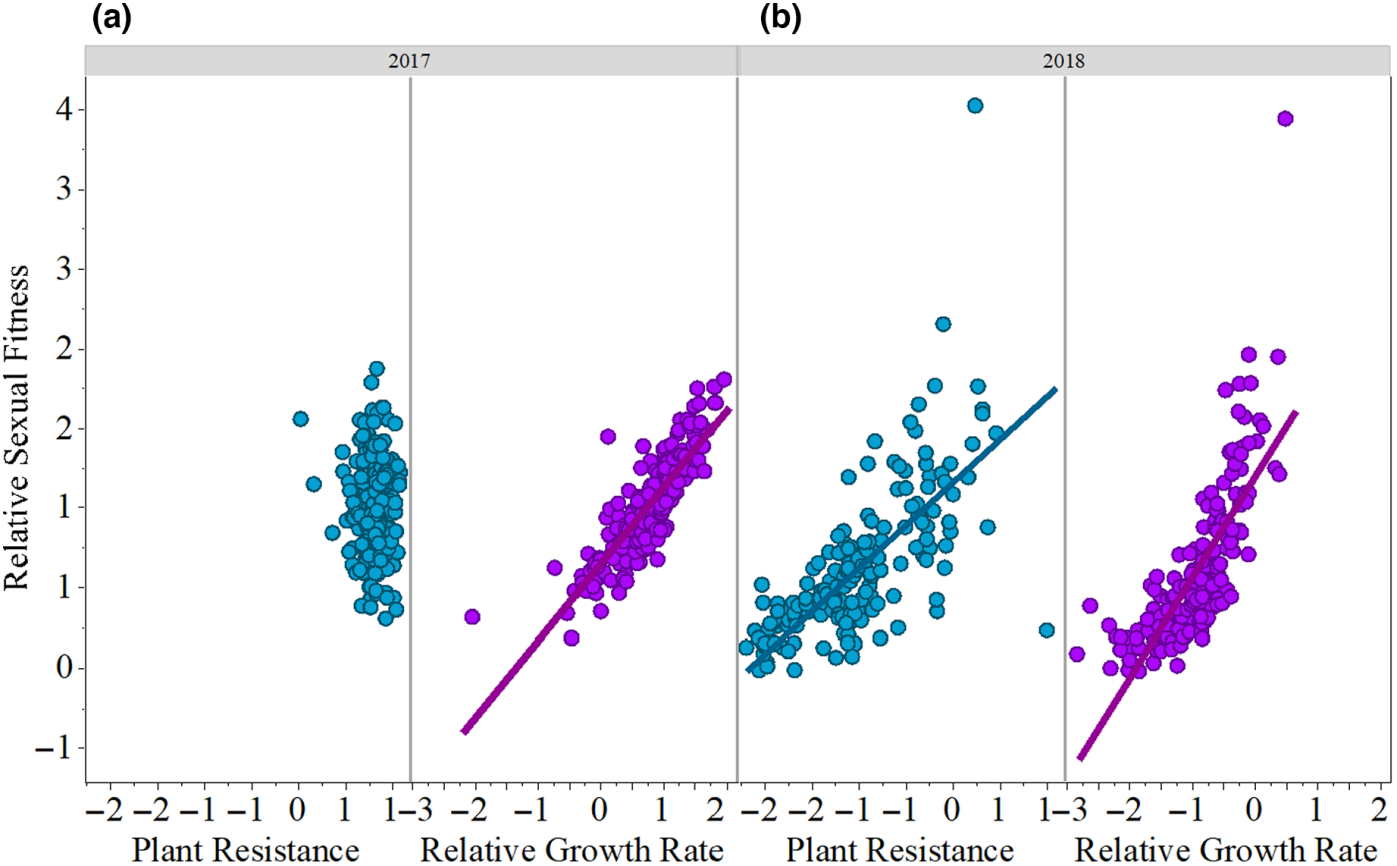
Relationships between relative sexual fitness, plant resistance, and relative growth rate in 2017 (a) and 2018 (b). Predictor variables were standardized to mean = 0 and standard deviation = 1. Lines of the generalized linear models are only shown for significant relationships (*p*‐value < .05). See also Table 2.

The GLMs of relative fitness versus resistance and RGR per year indicated that plants with increased resistance displayed higher fitness, albeit only in 2018 (Table 2, Figure 3a). Moreover, plants with higher relative growth rate also had higher fitness during both years (Table 2, Figure 3a). No significant interaction between RGR and resistance was found in both years (Table 2, Figure 3a).

**TABLE 2:** Relationships testing the effects of (a) resistance, (b) herbivore abundance (folivores), (c) seed predator (larvae of *Trichobaris soror*), and (d) relative growth rate (RGR) on relative sexual fitness in each year of study (2017 and 2018).

Response variable	Effects	*N*		df		*β*_*i*_		L‐R X^2^		*p*	
		2017	2018	2017	2018	2017	2018	2017	2018	2017	2018
(a) Sexual fitness	Resistance	213	164	3	3	−0.42 (0.35)	**0.55 (0.23)**	0.43	6.59	.5089	**.0102**
	RGR	213	164	3	3	**0.54 (0.07)**	**0.79 (0.24)**	61.12	11.17	**.0001**	**.0008**
	Resistance × RGR	213	164	3	3	1.06 (0.82)	0.10 (0.27)	0.91	0.11	.3384	.7366
(b) Sexual fitness	*Lema daturaphila*	212	167	9	9	**0.13 (0.04)**	−0.07 (0.10)	8.38	0.61	**.0038**	.4347
	*Trichobaris soror*	212	167	9	9	**0.09 (0.03)**	0.05 (0.10)	4.37	0.00	**.0365**	.9282
	*Epitrix parvula*	212	167	9	9	0.04 (0.03)	**0.29 (0.10)**	1.32	5.94	.2503	**.0147**
	Larvae of *Lema daturaphila*	212	167	9	9	**0.62 (0.10)**	**−0.85 (0.21)**	24.42	8.97	**.0001**	**.0027**
	RGR	212	167	9	9	**0.50 (0.05)**	**0.78 (0.22)**	76.68	14.26	**.0001**	**.0002**
	*Lema daturaphila* × RGR	212	167	9	9	−0.11 (0.08)	0.19 (0.18)	1.50	0.87	.2202	.3494
	*Trichobaris soror* × RGR	212	167	9	9	0.02 (0.08)	−0.11 (0.13)	0	0.37	1	.5417
	*Epitrix parvula* × RGR	212	167	9	9	−0.11 (0.07)	−0.17 (0.18)	1.78	0.74	.1815	.3886
	Larvae of *Lema daturaphila* × RGR	212	167	9	9	**−0.79 (0.21)**	0.28 (0.31)	8.63	0.50	**.0033**	.4769
(c) Sexual fitness	Larvae of *Trichobaris soror*	198	167	3	3	**0.23 (0.02)**	**−1.89 (0.63)**	69.93	10.78	**.0001**	**.0010**
	RGR	198	167	3	3	**0.53 (0.06)**	**0.92 (0.30)**	74.35	8.86	**.0001**	**.0029**
	Larvae of *Trichobaris soror* × RGR	198	167	3	3	**0.24 (0.04)**	**−2.84 (1.04)**	20.55	5.96	**.0001**	**.0146**

Abbreviations: df, degrees of freedom; L‐R X^2^, likelihood ratio test; *N*, number of individuals; SE, standard error; *β*_*i*_, selection gradients (generalized linear coefficients).

Significant *p*‐values are in bold.

**FIGURE 4:**
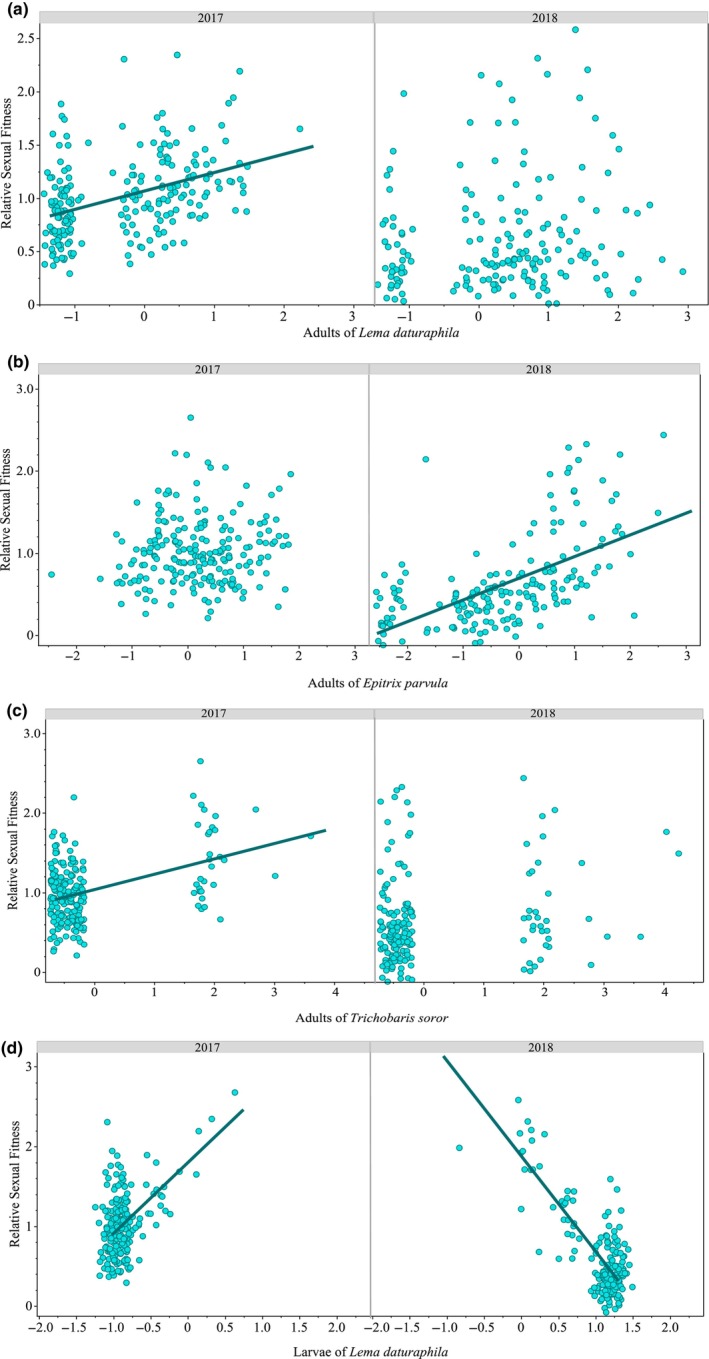
Relationships between relative sexual fitness, adults of *Lema daturaphila* (a), adults of *Epitrix parvula* (b), adults of *Trichobaris soror* (c), and larvae of *Lema daturaphila* (d) during 2017 and 2018. Predictor variables were standardized to mean = 0 and standard deviation = 1. Lines of the linear models are only shown for significant relationships (*p*‐value < .05). See also Table 2.

The GLMs of relative fitness versus herbivore abundance (both larvae and adults of *L. daturaphila* and *E. parvula*, and *T. soror* adults) revealed that plants exhibiting higher sexual fitness were associated with a greater abundance of the adults of *L. daturaphila* (only in 2017), *E. parvula* (only in 2018) and *T. soror* (only in 2017) (Table 2; Figure 4a–c). In 2017, larger and faster‐growing plants exhibited a decreased abundance of *L. daturaphila* larvae (*L. daturaphila* × RGR interaction; Table 2). In 2017, plants exhibiting greater sexual fitness were observed to have a higher abundance of *L. daturaphila* larvae. Conversely, in 2018, plants with higher sexual fitness displayed a lower abundance of *L. daturaphila* larvae (Table 2; Figure 4d). No significant interaction between herbivores and RGR was observed in 2018 (Table 2).

In 2017, larger and faster‐growing plants with higher fitness displayed a higher abundance of *T. soror* larvae (Table 2; Appendix 2). In contrast, in 2018, plants with higher fitness and growth rate also demonstrated a lower abundance of *T. soror* larvae (Table 2; Appendix 2).

## 4 DISCUSSION

In this study, during 2017, herbivory was significantly lower compared to 2018. In 2017, plant resistance did not increase plant fitness. In contrast, in 2018, the escalated herbivory caused mainly by *L. daturaphila* larvae led to higher plant mortality or reduced seed production in the surviving plants. However, these surviving plants with higher seed production also exhibited higher resistance and a lower abundance of *L. daturaphila* larvae. This suggests that plants allocated nutrient resources to resistance traits when faced with more severe herbivory in 2018. These findings emphasize the influence of *L. daturaphila* larvae as a selective agent of resistance in *D. stramonium* at the study site. Moreover, our findings also highlight how the year‐to‐year variation in the abundance of *L. daturaphila* may lead to frequency‐dependent selection for an increased plant resistance. For example, positive selection for plant resistance may be relaxed at lower abundance of *L. daturaphila* larvae. In contrast, outbreaks of this herbivore can increase the selective pressure, resulting in selection for an increased plant resistance, as we observed. Thus, conducting long‐term studies (e.g., >2 years) into plant‐herbivore interactions not only evaluates the consequences of species interactions but also considers them within the context of fluctuating population densities, changing environmental conditions, and shifts in the community composition (Agrawal & Maron, 2022).

Our results also demonstrated that larger plants with a faster growth rate exhibited higher fitness in both years. However, the effect size of the relative growth rate on fitness was more pronounced during the second year when the abundance of *L. daturaphila* larvae was higher. Furthermore, these larger plants with faster growth rates were also more resistant, particularly in 2018. This suggests that plants might have strategically allocated all their available nutrient resources to simultaneously resist herbivory and promote faster growth, thereby increasing their chances of survival under the intense herbivore attack experienced in 2018 (Allcock & Hik, 2004; Carmona & Fornoni, 2013; Fornoni et al., 2004). Previous research has shown that individuals with faster biomass accumulation have greater carbon availability, leading to increased growth of roots and shoots. This, in turn, improves access to light and soil nutrients, resulting in overall biomass accumulation and reaching the reproductive stage faster, thus ensuring their survival (Chiariello et al., 1989). Therefore, the increased resistance and RGR in 2018, may provide higher fitness benefits by simultaneously allocating resources to both traits. This synergistic strategy could surpass the advantages offered by either strategy alone when faced with severe herbivory (Carmona & Fornoni, 2013; Fornoni et al., 2004; Núñez‐Farfán et al., 2007; Stinchcombe & Rausher, 2002).

Our findings also contrast with the common growth‐defense trade‐off observed in resource allocation strategies of plants (Züst & Agrawal, 2017). While nutrient redirection to defensive traits often leads to a trade‐off between growth and defense (Ågren & Schemske, 1994; He et al., 2022; Herms & Mattson, 1992; Monson et al., 2022; Watts et al., 2023), certain plant species with high growth rates can still produce defensive traits, enhancing overall fitness (Almeida‐Cortez et al., 1999; Carmona & Fornoni, 2013). Our results suggest potential functional complementarity between resistance and growth traits, particularly when resistance alone is insufficient against herbivores. In such cases, a faster growth may help buffer the negative impact of herbivore damage on fitness (Carmona & Fornoni, 2013; Valverde et al., 2003).

We also observed that the damage caused by *L. daturaphila* larvae could potentially impact the fitness and survival of other specialist herbivores. For instance, in 2017, when plants were not subjected to lethal damage, they exhibited better health. Therefore, we recorded a higher abundance of *T. soror* (adults and larvae) and *E. parvula* in larger and faster‐growing plants that also had a higher fitness. In contrast, in 2018, a year marked by elevated plant damage by *L. daturaphila*, plants produced a lower average number of seeds and grew smaller compared to 2017, resulting in a decreased availability of seeds and leaves for *T. soror* and *E. parvula*. Therefore, the impact of *L. daturaphila* on plant fitness may intensify direct competition for limited plant resources. This is because *L. daturaphila* larvae consume considerable quantities of plant tissues, including leaves and seeds, thereby reducing resource availability for the other specialist herbivores.

During the second year of study, we also observed that plants displaying higher seed production exhibited a reduced abundance of *T. soror* larvae. We speculate that these plants might have defended themselves against this herbivore. Previous research has documented that the seeds of *D. stramonium* contain scopolamine, a tropane alkaloid known to serve as a defense against this seed predator (Miranda‐Pérez et al., 2016). Furthermore, in a prior study involving the same F_2_ progeny used here for 2018, we reported that scopolamine led to a decrease in the number of *T. soror* adults found on leaves (De‐la‐Cruz, Merilä, et al., 2020). These findings also stress the plants' capability to withstand attacks from multiple herbivores to survive (Iwao & Rausher, 1997; Juenger & Bergelson, 1998; Wise & Rausher, 2013).

Determining the causes of high plant damage—whether it arises from increased herbivore numbers, low plant resistance, or both—is complex. However, our correlation analysis did show that plants with more leaf damage had higher numbers of *L. daturaphila* larvae. Notably, this association was only observed during 2018, the year of the herbivore outbreak. Furthermore, in a previous study, we have reported the genetic basis for chemical defenses in the F_2_ progeny used here (De‐la‐Cruz, Merilä, et al., 2020). Our results revealed that plants with high leaf damage (low resistance) were more genetically related to the Teotihuacán parent, which was selected for its lower alkaloid concentration. Conversely, plants that exhibited low leaf damage (high resistance) were genetically more related to the Ticumán parent, selected by its higher alkaloid concentration (De‐la‐Cruz, Merilä, et al., 2020). Indeed, we observed positive selection for a triterpenoid compound in plants genetically related to the Ticumán parent (De‐la‐Cruz, Merilä, et al., 2020). This compound also reduced the abundance of *L. daturaphila* larvae in the F_2_ plants more genetically linked to the Ticumán parent (De‐la‐Cruz, Merilä, et al., 2020). It is noteworthy to mention that triterpenoids also inhibit the larvae of *Manduca sexta* in other Solanaceae species such as *Nicotiana tabacum* (Laothawornkitkul et al., 2008). Thus, our results indicate that plant resistance was inherited in the F_2_ progeny. We ruled out the likelihood of a phenotypic plastic response in plant resistance, as we would anticipate that plants more genetically related to the Teotihuacan parent (low resistance) would also exhibit high resistance, yet our observations did not show this (De‐la‐Cruz, Cruz, et al., 2020; De‐la‐Cruz, Merilä, et al., 2020).

In summary, our results are in line with our hypothesis that plant resistance traits reduce herbivory and confer a fitness advantage, particularly under the high levels of herbivory observed in the second year of the study. Our results did not support the hypothesized trade‐off between plant resistance, growth, and fitness. We anticipated that larger plants with accelerated growth would show higher fitness but reduced resistance to herbivores. However, our observations revealed no evidence of this trade‐off. Rather, it appears that plants allocated their nutritional resources to both resistance and growth as a survival strategy, especially under severe herbivore pressure from *L. daturaphila* larvae in 2018. This highlights how a major herbivore species (*L. daturaphila* larvae) can drive the evolution of plant defense mechanisms and, by competing for food resources, can influence the fitness of other herbivore species. Our study also highlights that fluctuating environments, exemplified by inter‐annual variations in herbivore abundance, have a significant impact on plant resistance and, ultimately, on plant performance.

## AUTHOR CONTRIBUTIONS

**Ivan M. De‐la‐Cruz:** Conceptualization (equal); data curation (equal); formal analysis (equal); investigation (equal); methodology (equal); project administration (equal); resources (equal); software (equal); validation (equal); visualization (equal); writing – original draft (equal); writing – review and editing (equal). **Juan Núñez‐Farfán:** Conceptualization (equal); funding acquisition (equal); investigation (equal); methodology (equal); project administration (equal); resources (equal); supervision (equal); validation (equal); writing – review and editing (equal).

## CONFLICT OF INTEREST STATEMENT

The authors declare that they have no competing interests.

## Data Availability

Data supporting the analyses of this manuscript can be consulted at https://doi.org/10.6084/m9.figshare.24305716.v1.

## APPENDIX 1

### 1.1

Correlograms illustrate the relationships between herbivore abundance and leaf area consumed (plant damage) and relative growth rate for the years 2017 (a) and 2018 (b). The magnitude and color intensity of each circle indicate the strength of the correlation. Only statistically significant correlations (*p*‐value < .05) are displayed; blank spaces denote non‐significant correlations.

## APPENDIX 2

### 2.1

Relationship between fitness, relative growth rate, and abundance of *Trichobaris soror* larvae across the 2 years of study (2017 and 2018). Predictor variables were standardized to mean = 0 and standard deviation = 1. Lines of the linear models are only shown for significant relationships (*p*‐value < .05). See also Table 2.
